# Investigation of the Oral Effects of Alcoholic Extract of Wild Yarrow (*Achillea wilhelmsii*) on Growth Performance, Immune, and Biochemical Serum Responses in Rainbow Trout (*Oncorhynchus mykiss*)

**DOI:** 10.1155/anu/2360780

**Published:** 2025-04-07

**Authors:** Maryam Shadmand, Amin Gholamhosseini, Azadeh Yektaseresht, Mahdi Banaee, Marzieh Heidarieh, Sara Bagheri, Mohammad Karimi, Milad Adel, Caterina Faggio

**Affiliations:** ^1^Division of Aquatic Animal Health and Diseases, Department of Clinical Sciences, School of Veterinary Medicine, Shiraz University, Shiraz, Iran; ^2^Department of Pathobiology, School of Veterinary Medicine, Shiraz University, Shiraz, Iran; ^3^Department of Aquaculture, Faculty of Natural Resources, Behbahan Khatam Alanbia University of Technology, Behbahan, Iran; ^4^Nuclear Science and Technology Research Institute, Tehran, Iran; ^5^Department of Aquatic Animal Health and Diseases, Iranian Fisheries Science Research Institute (IFSRI), Agricultural Research Education and Extension Organization (AREEO), Tehran, Iran; ^6^Department of Chemical, Biological, Pharmaceutical and Environmental Sciences, University of Messina, Messina, Italy

**Keywords:** *Achillea wilhelmsii*, biochemical parameters, growth performance, immune responses, rainbow trout

## Abstract

*Achillea wilhelmsii* (AW), a plant rich in flavonoids, including lutein, apigenin, rutin, and phenolic compounds with antioxidant, antibacterial, and antifungal properties, is used in traditional medicine. In this study, the impact of AW extract on the growth, immune response, and biochemical indices of rainbow trout (*Oncorhynchus mykiss*) was investigated. Over 8 weeks, fish were fed diets supplemented with varying concentrations of AW extract (0%, 0.5%, 1%, or 2%). No significant differences were observed in growth performance, glucose levels, or key enzymes such as lactic acid dehydrogenase, alanine transaminase, or aspartate aminotransferase (AST) between the AW-supplemented groups and the control group. However, fish that received AW supplementation showed significantly higher levels of total serum protein, lysozyme activity, superoxide dismutase (SOD) activity, and immunoglobulin M (IgM). Moreover, the AW-fed groups exhibited lower mortality after exposure to *Yersinia ruckeri*. In conclusion, AW supplementation could enhance immune function in rainbow trout and decrease mortality after exposure to *Y. ruckeri*. Therefore, using this plant (1% and 2%) in aquaculture could be justified as a means to increase resistance to pathogens and improve the immune system performance of fish.

## 1. Introduction

The aquaculture industry has grown significantly in recent decades due to the increasing global demand for sustainable protein sources. Rainbow trout (*Oncorhynchus mykiss*) is highly valued in aquaculture due to its impressive growth rate, resilience to stress, favorable taste, and economic viability, making it an ideal candidate for high-density aquaculture systems [1, 2]. Rainbow trout is one of the most important cold-water fish species farmed in Iran. Its adaptability to Iran's climatic conditions and favorable growth rate have contributed to its success in aquaculture. In addition, its high consumer acceptance has made it a valuable species for cultivation in intensive and semi-intensive aquaculture systems. According to statistics from the Iranian Fisheries Organization, its annual production is reported to be 200,000 tons. Thus, compliance with food hygiene, welfare conditions, and safety standards is essential to produce a healthy product [3]. Moreover, cultivation in crowded conditions can be stressful for fish. Exposure to stressors can suppress fish's immune systems and increase their susceptibility to infectious agents and pathogens. Furthermore, disease outbreaks in farmed fish remain a significant challenge, leading to significant financial losses and sometimes even bankruptcy for investors in this field [4, 5]. Aquaculture farmers have traditionally relied on antibiotics and chemical treatments to manage these diseases and reduce the associated challenges. Although these methods may be effective in the short term, their overuse raises concerns, including the emergence of drug-resistant pathogens, disruption of metabolic processes in fish, antibiotic bioaccumulation, and potential risks to human health [6, 7]. In addition, drugs and antibiotics used in aquaculture can pose environmental hazards.

Therefore, the search for alternative bio-based treatments has gained momentum in recent decades. Studies have shown that medicinal plants, probiotics, and immune stimulants can serve as promising solutions to replace antibiotics and chemicals. The use of these compounds can ensure fish health, immunity, and resistance to diseases without the side effects of conventional drugs [8, 9].

Studies show that herbal medicine has numerous benefits in aquaculture and can be a sustainable alternative to antibiotics and chemicals. Medicinal plants and their derivatives can enhance fish immunity, increase disease resistance, and reduce inflammation. Some medicinal plants and their phytochemical compounds have antimicrobial properties against pathogens. Herbal medicines reduce the risk of drug resistance, are environmentally friendly, and improve growth by enhancing digestion and nutrient absorption. In addition, they reduce chemical residues in fish products and ensure food safety. Affordable and locally available herbal medicine offers a practical solution to improve fish health and support sustainable aquaculture practices [5]. In addition to their antibacterial and anti-inflammatory properties, medicinal plants can enhance fish's immune systems, promote growth, and improve survival rates.

Several medicinal plants have been reported to enhance fish immunity and disease resistance. Dietary supplementation with *Oliveria decumbens* significantly improved antioxidant and antibacterial defenses against *Streptococcus iniae* in Nile tilapia, *Oreochromis niloticus* [10]. Similarly, *Eichhornia crassipes* leaf extract boosted immune parameters in rainbow trout, *O. mykiss* [11]. *Rhus verniciflua* exhibited antibacterial effects against *Edwardsiella tarda* in olive flounder, *Paralichthys olivaceus* [12]. Moreover, *Mentha longifolia* extract effectively inhibited *Yersinia ruckeri* in rainbow trout [13]. Additionally, *Lavandula angustifolia* supplementation in common carp, *Cyprinus carpio*, enhanced nonspecific immune parameters and reduced inflammation and oxidative stress [14]. Wang et al. [15] found that a Chinese herbal medicine mixture (CHMM) significantly improved the immune response !and resistance to infectious hematopoietic necrosis virus (IHNV) infection in rainbow trout by modulating immune gene expression and reducing oxidative stress markers [16]. Moreover, Pan et al. [17] reported that the administration of purified Astragalus polysaccharide (P-APS) as an adjuvant increased vaccination efficacy against IHNV in rainbow trout. Similarly, Yilmaz et al. [18] reported that *Valeriana officinalis* and *Passiflora incarnata* extracts could modulate hematological and biochemical parameters following exposure to *Lactococcus garvieae*. Pourmand et al. [19] found that dietary supplementation with garlic, black seed, and black caraway essential oils enhanced immune parameters in rainbow trout, with garlic essential oil showing the most pronounced effects, particularly in boosting lysozyme activity, complement levels, and TNF-*α* expression.

Medicinal plants play a crucial role in mitigating oxidative stress, a major challenge in intensive aquaculture systems. Ghafarifarsani et al. [20] reported that a herbal mixture of *Malva sylvestris*, *Origanum vulgare*, and *Allium hirtifolium* improved antioxidant enzyme activity (superoxide dismutase (SOD), CAT, GR), thereby, reducing oxidative damage in common carp. Similar effects were observed with *Juniperus communis* essential oil (JCEO) in rainbow trout [21], indicating the broad applicability of plant-based antioxidants.

Medicinal plants contribute to improved fish growth and feed efficiency. Aghili et al. [22] found that dietary supplementation with turmeric, *Curcuma longa*, and black pepper, *Piper nigrum*, enhanced growth, hematological parameters, and immune function in rainbow trout. Köse, Karabulut, and Er [23] showed that *Taraxacum officinale* extract improved protein efficiency and growth in rainbow trout. Similarly, Kaya, Karataş, and Guroy [24] found that pot marigold, *Calendula officinalis*, enhanced growth indices and feed conversion efficiency.

The incorporation of medicinal plants in aquaculture offers eco-friendly and cost-effective alternatives to synthetic drugs and antibiotics. Studies have shown that medicinal plants used as dietary supplements can improve aquaculture outcomes [2, 25–27]. Semwal, Kumar, and Kumar [28] reported the sustainability of plant-based treatments, promoting organic aquaculture while minimizing environmental impact. Their widespread availability and low cost make them viable options for improving fish health and production efficiency.

Among the many medicinal plants being explored for their potential in aquaculture, *Achillea wilhelmsii* (AW), stands out due to its well-documented therapeutic properties. Wild or overgrown yarrow (AW), a member of the chicory family, is widely distributed in the northern regions of Iran, including Mazandaran and Golestan provinces [29], as well as Azerbaijan, Fars, Sistan and Baluchistan, Hamedan, and Tehran provinces. This small herbaceous plant (15–40 cm) has a branched stem and green and hairy leaves.

Over 100 biologically active compounds have been identified in yarrow [30]. Yarrow (AW) is a traditional herbal medicine known for its anti-inflammatory, antimicrobial, and antioxidant properties, primarily due to its flavonoid compounds, such as lutein, apigenin, rutin, and phenolic compounds [31]. This plant extract has improved immune function in various animals, including fish [32]. Akbar et al. [33] showed that the ethanolic extract of *A. santolinoides* subsp. *wilhelmsii* (WEEAW) had potent antioxidant, antimicrobial, and anti-inflammatory effects, particularly against *Staphylococcus aureus*. The essential oil of AW, rich in fragranol, fragranyl acetate, and oleic acid, demonstrated notable antimicrobial activity, particularly against *Candida albicans* and *S. aureus*, as well as effectiveness against gram negative bacteria like *Acinetobacter baumannii* and *Shigella dysenteriae* [34]. Sirakov et al. [35], Bahabadi et al. [32], and Koshinski [36] found that yarrow supplementation improved growth and blood biochemical parameters in rainbow trout. Alinezhad [37] showed yarrow extract improved hematological and immune responses in common carp, enhancing red blood cell (RBC) counts, immunoglobulin (Ig) levels, and lysozyme concentration. Banaee et al. [38] demonstrated that yarrow extract reduced mortality and improved enzyme activities in carp infected with *Aeromonas hydrophila*. Altinterim, Danabas, and Aksu [39] found that yarrow hydrosol improved hematological and immune parameters in common carp infected with *Y. ruckeri*, suggesting its potential in aquaculture.

Therefore, the use of immune system stimulants such as plant derivatives may improve fish's immune system function and support their health in intensive culture systems [40]. In this study, it was hypothesized that the use of yarrow (AW) extract can improve the immune system function and physiology of rainbow trout (*O. mykiss*). This study aimed to evaluate biochemical, immune, and oxidative stress indices in fish treated with different concentrations of yarrow.

## 2. Material and Methods

### 2.1. Fish

Three hundred and seventy-one pieces of rainbow trout, weighing an average of 14.7 ± 1.7 g, were purchased from the Delkhan Fish Breeding Workshop in Sepidan County (Fars Province, Iran). Experimental protocols were performed in accordance with the Iranian animal ethics framework under the supervision of the Iranian Society for the Prevention of Cruelty to Animals and Shiraz University Research Council (IACUC no: 9132738). According to standard protocols, the fish were transferred to the laboratory in aerated tanks. Before introducing them to the laboratory, the halls and storage tanks disinfection was carried out using a formalin dose of 250 ppm for 60 min, and the 500-L tanks were dewatered to prevent contamination. The fish were distributed into 12 groups at random, with four treatments, and each treatment included three replicates. Each replicate consisted of 25 fish, ensuring sufficient numbers for statistical analysis. The water in all 12 tanks was changed by 20% daily to maintain optimal water quality. Fish were adapted for 3 weeks to their new environment before any experimental treatments were applied. The well was used as the water source. Throughout the test period, water parameters were closely monitored, and the temperature was maintained at 14.7 ± 1.9°C, the pH level was 7.4 ± 0.33, the dissolved oxygen (DO) content was 6.5 ± 1.09 mg/L, the total suspended solids (TSSs) was 29 ± 0.15 mg/L, total dissolved solids (TDSs) was 450 ± 50 mg/L, electrical conductivity (EC) was <200 µS/cm, ammonia (NH_3_/NH_4_^+^), nitrite (NO_2_^−^), and nitrate (NO3^−^) were <0.05, <0.2, and <25 mg/L, hardness and alkalinity was 150 ± 25 mg/L as CaCO_3_. These conditions were carefully regulated to promote the health and welfare of the fish during the study. Measuring water quality, including temperature, TSS, TDS, EC, pH, DO, ammonia, nitrite, nitrate, hardness, and alkalinity, was done with appropriate meters and test kits, such as a thermometer, pH meter, DO meter, and colorimetric kits for ammonia and nitrites.

### 2.2. AW Extract Preparation, Feed Formulation, and Administration

Wild yarrow (AW) was collected from the natural habitat of this plant in the Fars Province (Faculty of Agriculture). The extraction was prepared according to Gholamhosseini et al. [41]. Briefly, 10 kg of wild yarrow leaves were pulverized in a dry environment, away from sunlight, in dry air, using a mill [41]. Following the method described by Gholamhosseini et al. [41], the obtained powder was mixed in 1-L of 80% methanol in a ratio of 1:5 in flasks. The mixture was gently agitated on a shaker for 48 h to facilitate extraction. The resulting mixture was then filtered through a strainer and Buchner funnel. The solvents were then evaporated from the primary extracts using a rotary evaporator to yield the concentrated yarrow extract [27].

A preliminary study was conducted to determine the optimal concentration range for wild yarrow (AW) extract. The selected concentrations were 0.25%, 0.5%, 1%, 2%, 3%, and 4% of wild yarrow extract mixed with the basal diet. Based on these findings, four experimental diets were formulated with 0% (control group), 0.5%, 1%, and 2% of the extract. Each supplemented diet was entirely combined with water to form a consistent paste, which was then processed using a Pars Khazar meat grinder. The paste was extruded at a mechanical pressure of 200 kPa using an extruder cylinder, followed by drying in warm air for 24 h. The dried product was then ground into pellets with an approximate diameter of 0.3 cm and stored at refrigeration temperatures. Throughout the 8-week study, the fish were fed three times daily, with the feeding amount set at 2% of their body weight (Table 1).

### 2.3. Sampling Time and Method

After 56 days, 15 fish from each treatment group (five per iteration) were randomly sampled to evaluate growth and immunochemical responses. Feeding was stopped for 24 h before sampling to standardize the fish's physiological state and minimize any potential confounding effects from recent feeding. After sampling, the challenge was evaluated.

### 2.4. Growth Performance

The growth performance of the fish was measured using the following formula [41]. Moreover, to assess growth performance, fish were starved for 24 h before sampling and weighing to ensure accurate measurements. The growth performance parameters were calculated based on initial and final measurements. Specific growth rate (SGR) was determined to measure growth rate as a percentage per day, using the formula:

  $$\begin{array}{c} \text{SGR}=\frac{\ln W_{f}-\ln W_{i}}{T}\times 100, \end{array}$$

where *W*_*f*_ is the final weight, *W*_*i*_ the initial weight, and *T* the duration of the study in days.

Weight gain (WG) was calculated as the difference between final and initial weights:

  $$\begin{array}{c} \text{WG}=W_{f}-W_{i}. \end{array}$$

Feed conversion ratio (FCR) was assessed to determine feed utilization efficiency, calculated as:

  $$\begin{array}{c} \text{FCR}=\frac{\text{Feed consumed}\left(\text{g}\right)}{\text{Weight gain}\left(\text{g}\right)}. \end{array}$$

Finally, condition factor (CF) was evaluated to relate fish weight to length, providing an indicator of body condition, using the formula:

  $$\begin{array}{c} \text{CF}=\frac{W}{L^{3}}\times 100, \end{array}$$

where *W* represents weight in grams and *L* is the length in centimeters.

### 2.5. Blood Collection

Blood sampling was conducted following capture and anesthesia using clove powder at a concentration of 150 ppm. Blood samples were collected from the caudal vein with a 3-cc syringe, utilizing sterile, nonheparinized tubes for biochemical and immunological analyses. In contrast, heparinized tubes were used to assess hematology indicators. The nonheparinized samples were centrifuged at 6000 rpm for 10 min at 4°C to separate the serum. The serum samples were promptly stored in a freezer at −80°C, as recommended by Banaee, Sureda, and Faggio [42].

### 2.6. White Blood Cell (WBC) and RBC Count

Nat and Herrick staining methods were used to count WBC and RBC. One milliliter of blood was mixed with a staining solution to achieve a final volume of 50 mL. This mixture was incubated for 30 min at room temperature to allow for effective staining. After incubation, the cell count was performed using a calibrated hemocytometer slide. The numbers of WBCs and RBCs were calculated and expressed as cells per microliter (cells/µL) [43].

### 2.7. Serum Biochemical Markers

The assessment of biochemical parameters, including glucose, total protein, triglycerides, cholesterol, liver enzymatic activities (lactate dehydrogenase (LDH), alanine aminotransferase (ALT), and aspartate aminotransferase (AST)), and creatinine, was conducted using Pars Azmoon commercial kits [27, 44]. Each biochemical parameter was assessed according to the specific instructions provided with the Pars Azmoon kits, using appropriate volumes of serum and reagents. All biochemical parameters were measured by an autoanalyzer (Sanjesh Co. Iran).

### 2.8. Oxidative Biomarkers

The assessment of SOD activity was conducted using the SD125 Ransod kit (RANDOX, UK). The reaction mixture was prepared according to the manufacturer's instructions, combining the serum with Xanthine Oxidase and 2-(4-iodophenyl)-3-(4-nitrophenol)-5-phenyl tetrazolium chloride (I.N.T). This mixture was incubated at 37°C for a specified duration. After incubation, the absorbance of the reaction mixture was measured at approximately 490 nm using a spectrophotometer. The SOD activity was calculated based on the level of inhibition of the reaction, comparing the absorbance of the sample to that of the control, which contained no sample [45].

### 2.9. Immunological Parameters

Serum lysozyme activity was quantified using the method described by Parry, Chandan, and shahani [46]. A suspension of *Micrococcus lysodeikticus* cells in 0.05 M sodium phosphate buffer (pH 6.2) at a concentration of 0.2 mg/mL was prepared as the substrate for lysozyme. For the assay, 25 μL of serum was added to a cuvette containing 2.5 mL of the *M. lysodeikticus* cell suspension and the absorbance was immediately measured at 450 nm using a spectrophotometer. The decrease in absorbance at 450 nm, recorded over 1–3 min at 15-s intervals, indicated lysozyme activity as the enzyme degrades bacterial cell walls, reducing turbidity. Lysozyme activity was calculated as the rate of decrease in absorbance per minute, with activity typically expressed in units per milliliter (U/mL), where one unit represents a decrease in absorbance of 0.001 per min. A blank sample, using sodium phosphate buffer instead of serum, was included to control for nonenzymatic changes in absorbance [41].

Serum IgM levels were measured using the immunoturbidimetry method with a commercial kit (Pars test). This kit typically includes a sample diluent for serum preparation, a primary reagent (Solution No. 1) containing specific antibodies that bind to IgM and form immune complexes, and a secondary reagent (Solution No. 2) that enhances sensitivity by binding to these complexes. In a 96-well microplate, Solution No. 1 was added to all wells, mixed, and incubated at 37°C for 3–5 min. The initial absorbance of the serum samples and standards was measured at 340 nm. Solution No. 2 was then added, mixed, and incubated at 37°C for an additional 10 min before measuring the absorbance again. The change in absorbance was calculated by subtracting the initial from the secondary absorbance, and the IgM concentration in the serum samples was determined using a standard curve generated from the provided standards.

### 2.10. Bacterial Challenge

The method described by Gholamhosseini et al. [41] was followed to investigate the survival rate of rainbow trout (*O. mykiss*) after a challenge with *Y. ruckeri* (KC291153). Initially, a bacterial culture of *Y. ruckeri* was grown in tryptic soy broth (TSB) at 22°C for 24 h and then, adjusted to the desired concentration (e.g., 1 × 10^7^ CFU/mL) using sterile phosphate-buffered saline (PBS), with the concentration confirmed by plating on tryptic soy agar (TSA). Next, fish in each experimental group were exposed to the bacterial suspension through either intraperitoneal injection (0.1 mL of 1 × 10^7^ CFU/mL) or immersion, while control fish received sterile PBS. After the challenge, the fish were monitored daily for clinical signs of infection, behavioral changes, and mortality, with water quality parameters consistently maintained. Mortality data were recorded for each group over a specified period (e.g., 14 days postchallenge), allowing for the calculation of survival rates as a percentage of surviving fish out of the initial total. Cumulative mortality rates were also determined to compare responses between groups. Dead fish were removed immediately to prevent further disease spread and the remaining population was closely monitored for signs of infection. Dead fish were immersed in lime water to exterminate.

### 2.11. Statistical Analysis

Data analysis was performed using SPSS software version 18. Initially, the Shapiro–Wilk test was applied to evaluate the normality of the data distribution. Following this, a one-way analysis of variance (ANOVA) was conducted to determine the overall differences between treatment groups. Next, Duncan's multiple range test was employed for post hoc comparisons to identify specific group differences (*p* < 0.05). To minimize experimental error, all measurements were conducted in triplicate.

## 3. Results

### 3.1. Fish Growth

In this study, the inclusion of AW in the diet did not result in significant alterations in WG, SGR, or FCR when compared to the control group (Table 2).

### 3.2. Haematological Parameters

The hematological findings for fish that received different concentrations of AW are presented in Table 3. All treatment groups demonstrated significant increases in RBC, hematocrit (Htc), and hemoglobin (Hb) relative to the control group. Significant differences in WBC counts were observed only in the 1% and 2% AW groups compared to the control. Feeding fish with AW extract showed significant increases in Hb and Htc levels (*p*  < 0.05). The 1% AW diet group showed a significant decrease in circulating lymphocytes, while neutrophil counts significantly increased. No significant differences in monocyte percentages were detected between the groups (*p*  > 0.05; Table 3).

### 3.3. The Blood Biochemical Parameters

The blood biochemical markers for groups supplemented with AW over 8 weeks are summarized in Table 4. Glucose levels remained comparable between the supplemented groups and the control group (*p*  > 0.05). Creatinine concentrations were lower in the supplemented groups than in the control, with a statistically significant reduction observed specifically in the 1% AW group (*p*  < 0.05). No significant differences were found in the levels of AST, ALT, or LDH among the rainbow trout, *O. mykiss*, after 8 weeks of AW supplementation, with the 1% AW group showing the lowest values. Furthermore, lipid-related parameters, including cholesterol and triglycerides, were reduced following AW administration, especially in the 0.5% and 1% AW groups (Table 4). Serum protein levels increased in the groups receiving AW extract, with the most pronounced elevation observed in the 1% and 2% AW groups.

### 3.4. Immunological Parameters

The 1% AW treatment group exhibited a significant enhancement in lysozyme activity compared to the control group, with a *p*-value of less than 0.05. Additionally, AW-supplemented diets led to a notable rise in serum immunoglobulin M (IgM) levels, particularly in the 3% AW treatment group (Table 5).

### 3.5. Oxidative Biomarkers

**Figure 1:**
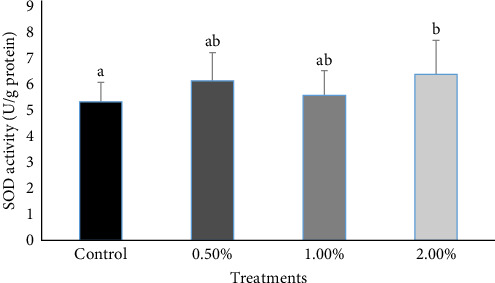
The mean superoxide dismutase (SOD) levels in rainbow trout fed varying amounts of *Achillea wilhelmsii* (AW) for 8 weeks are illustrated in the graph. Data are expressed as mean ± standard deviation. Significant differences (*p*  < 0.05) within the same row are denoted by different superscript letters.

In this study, the levels of SOD were found to increase in the groups given different doses of AW extract, with the 2% AW group exhibiting significantly higher enzyme levels than the others (Figure 1). Furthermore, using 1% and 2% AW extracts increased humoral immune parameters, including IgM, with the highest levels of IgM observed in the 2% group after 8 weeks.

### 3.6. Serum Bactericidal Activity

The bactericidal activity of the serum was tested against *S. iniae*, *Y. ruckeri*, *L. garviea*, and *A. hydrophila* bacteria after the eighth week, and the fish fed with 2% AW extract showed the best performance against *A*. *hydrophila*, and the 1% and 2% groups against *Y. ruckeri* (Table 6).

### 3.7. Bacterial Challenge

**Figure 2:**
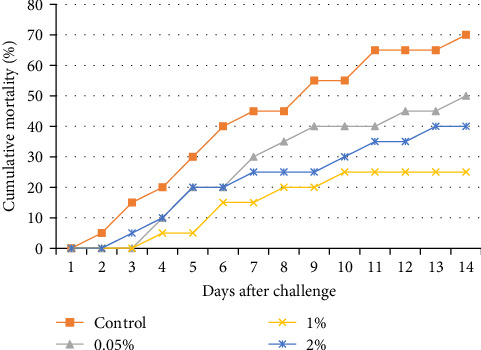
Cumulative mortality of treatments after challenged with *Yersinia ruckeri*.

After the analysis, all treatment groups were challenged with *Y. ruckeri*, a pathogen that poses a significant threat to aquaculture operations (Figure 2). Notably, the cumulative mortality rate for all of the AW treatment groups was substantially lower than that of the control group, and the 1% AW-supplemented group experienced the lowest mortality rate.

## 4. Discussions

### 4.1. Growth Performance

Studies have shown that medicinal plant extracts can improve fish growth and feed efficiency. In this research, dietary supplementation with AW did not significantly alter WG, SGR, or FCR when contrasted with the control group (Table 2). Improvement of growth performance and reduction of food conversion ratio in fish can be due to the favorable effects of medicinal plants on digestion, stimulation of bile secretion, and improvement of pancreatic enzyme function [47]; however, these results were not observed in previous studies. Ghafarifarsani et al. [20] found increased digestive enzyme activity in carp, indicating improved nutrient absorption. Köse, Karabulut, and Er [23] found that dandelion root extract improved growth and protein efficiency in rainbow trout. Kaya, Karataş, and Guroy [24] reported that pot marigold extract enhanced growth indices and feed conversion in rainbow trout. Wild yarrow plants (AW) contain several compounds, including flavonoids, saponin, and terpenes, which are related to the antibacterial properties of this plant. In addition, this plant can make food more palatable because of its attractive compounds, which provide a sense of smell. Studies have shown that using plant derivatives with antibiotic properties could improve carcass quality and growth by changing and enhancing the flora of the gastrointestinal system. Moreover, some compounds in medicinal plants, including flavonoids, increase fish's growth performance and health [8]. Increases in the FW, WG, and SGR of yellow catfish, *Pelteobagrus fulvidraco*, after feeding with *Glycyrrhiza uralensis* extract have been reported [48]. In addition, significant increases in WG, SGR, and FW have been observed in *O. niloticus* fed gotu kola, *Centella asiatica* powder [49]. In another study, improvements in SGR and FCR in common carp, *C. carpio*, were observed owing to the use of different doses of polyphenols extracted from chestnut and olive mill wastewater [50]; however, these results are in contrast to those of previous studies. The 8-week feeding trial demonstrated that garlic powder supplementation significantly enhances growth performance in juvenile rainbow trout, *O. mykiss*, with higher SGR and improved FCR compared to probiotics and mixed treatments [51]. Ghafarifarsani et al. [20] found that a herbal mixture of *M. sylvestris*, *O. vulgare*, and *A. hirtifolium* significantly enhanced growth and reduced FCR in common carp. Hajirezaee et al. [21] observed similar growth improvements in rainbow trout with common juniper, *J. communis*, essential oil supplementation.

### 4.2. Hematology

Hematological markers are crucial indicators for evaluating fishes' physiological well-being and health, as they provide valuable insights into various physiological processes [52, 53]. Nutritional factors and dietary supplements can affect blood cells, and the impact of these factors depends on the different types of leukocytes [44]. Feeding fish with diets containing AW extract resulted in increased levels of RBCs, Hb content, and Htc. At the same time, the total count of WBC displayed significant changes only in the 1% and 2% AW groups compared to the control group (Table 3). Although there was no significant difference in the number of blood monocytes, a remarkable increase in the number of neutrophils was observed in the 1% AW treatment group compared to the control group. The increase in RBC, Hb, and Htc levels suggests that AW extract may enhance oxygen transport and improve fish health, potentially due to its antioxidant or immunomodulatory properties. Significant changes in WBC counts were dose-dependent, indicating an effect on immune activation. A considerable increase in neutrophils suggested that the 1% extract may enhance the fish's innate immune response, improving infection resistance and stress. Similarly, administering tarragon and mint extracts orally enhanced the RBC count, WBC count, Hb content, and Htc levels in rainbow trout, *O. mykiss*, and Caspian whitefish, *Rutilus kutum* [41, 44]. Güroy et al. [51] found that garlic supplementation significantly affected hematological parameters [51]. Aghili et al. [22] reported that the inclusion of turmeric, *C. longa*, and black pepper, *P. nigrum*, in the diet enhances growth performance and improves hematological parameters in rainbow trout. Similarly, Yilmaz et al. [18] found that administering extracts from *V. officinalis* and *P. incarnata* adjusts hematological and biochemical parameters in rainbow trout following exposure to *L. garvieae*.

### 4.3. Biochemical Indicators

Blood biochemical parameters can be relied upon to assess the health status of fish accurately. These parameters change in response to changes in nutritional status, making them valuable for determining the well-being of fish [54, 55]. In this study, the serum glucose levels of fish treated with the AW extract were not significantly different from those of the control group. Previous studies have shown that flavonoids in plants can reduce intestinal glucose absorption by inhibiting the sodium-dependent glucose transport mechanism [56]. However, this has not been observed in this study. Creatinine is a byproduct of normal muscle metabolism that is typically produced consistently, filtered by the kidneys, and excreted in the urine. Its level reflects renal filtration, and creatinine indicates kidney health [57]. Any kidney damage can increase creatinine levels [58]. The results of this study revealed that the creatinine levels in the control group were higher than those in the treatment group. Therefore, the reduction in blood creatinine in fish treated with the AW extract indicates an improvement in the efficiency of the renal system in creatinine excretion. The activities of AST, ALT, and LDH did not significantly change in the serum of rainbow trout after 8 weeks of feeding with different doses of AW extract compared to the control group and the lowest values were observed in the 1% group. SGOT or AST is a ubiquitous enzyme present throughout the body, particularly in the heart and liver, and to a lesser extent in the kidneys and muscles. As an essential enzyme in the liver, AST plays a crucial role in neutralizing toxic agents and participating in metabolic activities. On the other hand, ALT, a glycoprotein enzyme attached to the cell membrane, is involved in various cellular processes, including growth and migration, protein phosphorylation, and apoptosis [54, 55]. Additionally, LDH is an enzyme involved in cellular respiration and is released into the stream during liver disease, anemia, and muscle damage [59]. Interestingly, Adangale, Ghosh, and Wairkar [60] found that the thyme-fed group had significantly lower LDH levels, indicating stable liver and biliary function in response to Cd during the experiment. The total protein concentration in plasma compared to the standard range serves as a clinical index to assess aquatic organisms' health, stress, and physiological condition [45]. Plasma proteins, primarily produced in the liver cells, are essential extracellular proteins that reflect the liver's nutritional status and proper functioning [57]. According to Ahmadi et al. [61], there is a direct correlation between the total plasma protein concentration and the levels of hepatic protein synthesis. Consequently, the increase in total plasma protein observed in rainbow trout fed 1% and 2% AW extract can be attributed to improved liver protein synthesis. The same study also revealed that adding plants like mistletoe, *Viscum album*, stinging nettle, *Urtica dioica*, ginger, *Zingiber officinale*, silymarin, *Silybum marianum*, and yarrow extract, *A. millefolium* [27, 32, 55], to fish diets increased total plasma protein levels. A significant change in total protein was observed in the serum of rainbow trout, *O. mykiss*, fed turmeric, *C. longa*, and black pepper, *P. nigrum*, supplements [22].

In contrast, triglyceride and cholesterol levels decreased in fish fed diets containing AW extract compared to the control group, especially in the 0.5% and 1% treatments. Triglyceride levels indicate nutritional status and serum total protein and cholesterol levels [62]. Flavonoids in plants have been shown to decrease blood cholesterol levels by affecting the metabolic reactions of different tissues in fat metabolism [63]. As a result, the hepatoprotective, hypoglycemic, antiplatelet, and antioxidant properties of yarrow make it a promising candidate for use as a hypolipoproteinemic and antiatherosclerotic agent [64]. Hajirezaee et al. [21] showed that feeding rainbow trout with common juniper, JCEO, increased total protein levels while reducing ALP and ALT activities.

### 4.4. Immunological Parameters

Medicinal plants are effective immunostimulants. Lysozyme plays a critical role in fish immune systems, acting as a defense mechanism against invading microorganisms and bolstering resistance [6, 65]. In this study, serum lysozyme activity significantly increased in fish fed diets containing 0.5% and 1% AW extract compared to the control group. Serum lysozyme activity increased significantly in the 0.5% and 1% AW extract groups, indicating improved innate immunity. Lysozyme breaks glycosidic bonds in bacterial walls, disrupting their normal function [66]. Hajirezaee et al. [21] found that the administration of common juniper, JCEO, could increase lysozyme activity, alternative complement activity (ACH50), and total Ig levels in rainbow trout, as well as improve survival rates after a challenge with *Y. ruckeri*. Wang et al. [15] showed that the CHMM could enhance the immune response and resistance to IHNV infection in rainbow trout. Pan et al. [17] reported that P-APS as an adjuvant increases vaccination efficacy against IHNV in rainbow trout. Moreover, Pourmand et al. [19] showed that dietary supplementation with garlic, black seed, and black caraway essential oils improves immune parameters in rainbow trout, with garlic exhibiting the most potent effects on lysozyme activity, complement levels, and TNF-*α* expression.

The results showed that AW extract enhances the fish's immune system. Higher doses enhanced IgM values, particularly in the 2% AW group, suggesting a boost in humoral immune response and pathogen resistance. Research by Adel et al. [44] has also shown that dietary administration of medicinal plants can enhance the overall health of rainbow trout by boosting innate immune responses. Ghafarifarsani et al. [20] reported increased levels of immune markers like Ig and lysozyme in common carp.

### 4.5. Oxidative Biomarkers

Oxidative stress can impair fish health, especially in intensive systems. The significant increase in SOD activity in fish fed with AW extract showed improved antioxidant defense. SOD is a critical antioxidant enzyme that safeguards cells from reactive oxygen species (ROS) and free radical chain reactions [67]. It catalyzes the conversion of superoxide radicals into less harmful hydrogen peroxide [68], thereby, bolstering the cellular defense mechanisms against ROS [69]. The highest SOD levels in the 2% AW group indicated a dose-dependent effect, likely due to the extract's bioactive compounds, such as flavonoids and phenolics. These findings showed that AW may improve fish resilience to oxidative stress, potentially enhancing overall health and stress tolerance. Additionally, antioxidants such as vitamin E, coenzyme Q, vitamin C, glutathione, and selenium function synergistically to mitigate lipid peroxidation and cellular damage [70]. Plant polyphenolic compounds act as electron donors, exerting antioxidant effects through enzymatic and nonenzymatic pathways. Yarrow extract demonstrated antioxidant properties by reducing the accumulation of malondialdehyde and salicylic acid, indicating its ability to prevent lipid peroxidation [25]. Hajirezaee et al. [21] demonstrated that the inclusion of common juniper, JCEO, in the diet of rainbow trout resulted in elevated CAT and SOD activities. Wang et al. [15] found that the CHMM could improve the functions of antioxidant and immune-related enzymes while reducing oxidative stress markers and modulating immune gene expression. Ghafarifarsani et al. [20] found that medicinal plants increased antioxidant enzyme activities (SOD, CAT, and GR) in fish, reducing oxidative damage and improving overall health. Flavonoids, natural antioxidants found in plants, enhance blood circulation, reduce inflammation, and inhibit cancer cell growth [71].

### 4.6. Survival After the Challenge

Medicinal plants improve immunity and disease resistance. Moreover, medicinal plants offer potential antimicrobial benefits. Plant extracts rich in bioactive compounds, such as flavonoids and alkaloids, offer an eco-friendly alternative to antibiotics. After the challenge with *Y. ruckeri*, the 1% group exhibited the lowest cumulative frequency of casualties, which was considered a significant achievement. Waine et al. [72] established that the onset of susceptibility to *Y. ruckeri* infection in rainbow trout occurs at 14 days posthatch (dph). They showed significant variations in mortality rates exist across different developmental stages, indicating a critical period for managing ERM in juvenile salmonids. In exploring the mechanisms of *Y. ruckeri* infection in fish, Yang et al. [73] provided insights into the inflammatory responses and alterations in intestinal microbiota composition resulting from the infection. Additionally, Soto-Dávila et al. [74] reported a significant upregulation of pro-inflammatory cytokines during natural infections with *Y. ruckeri*. A study by Harikrishnan et al. [75] showed that the injection of extracts from three plants—neem, *Azadirachta indica*, basil, *Ocimum sanctum*, and turmeric, *C. longa* led to a significant increase in fish resistance and immune response in goldfish, *Carassius auratus*. Terzi et al. [76] showed that *Prunus domestica* extract enhanced immune responses and survival in rainbow trout infected with *Y. ruckeri*. Similarly, Hajirezaee et al. [21] reported increased survival rates in juniper-supplemented trout challenged with the same pathogen, suggesting a natural alternative to antibiotics for disease prevention. Moreover, Yang et al. [77] highlighted the potential of dietary interventions in enhancing fish health, demonstrating that dietary supplementation with *Agaricus bisporus* polysaccharides (ABPs) significantly improved growth, immune response, and antioxidant activity in channel catfish, *Ictalurus punctatus*, challenged with *Y. ruckeri*. Adeli, Shamloofar, and Akrami [78] showed that lemon verbena, *Aloysia triphylla*, extract boosted immune parameters and reduced stress in trout. Köse, Karabulut, and Er [23] found dandelion root extract improved survival rates against *L. garvieae* infection.

## 5. Conclusion

The findings of this study indicated that diets supplemented with AW may have beneficial effects on the blood and biochemical parameters, as well as enhancing the immune response of rainbow trout. Although no significant improvements in growth performance, such as WG, SGR, or FCR, were observed, the inclusion of AW resulted in beneficial effects on blood profiles, with increased RBC counts and improved kidney function indicated by lower creatinine levels. Moreover, the study revealed enhancements in serum lysozyme activity and SOD levels, emphasizing the immune-boosting properties of AW. Thus, based on these results, feeding rainbow trout a diet supplemented with 2% AW is recommended. Future studies are encouraged to explore the effects of AW under intensive culture conditions to validate these findings and to understand its potential applications in aquaculture.

## Figures and Tables

**Table 1:** The experimental diet's formula and approximate composition.

Component	Quantity (g per 100 g)			
Corn Starch	4.9	4.9	4.9	4.9
White wheat flour	4.9	4.9	4.9	4.9
Soybean meal	25	25	25	25
Fish oil	5.5	5.5	5.5	5.5
Fish meal	45	45	45	45
Gelatin by-product	2	2	2	2
Oil of canola	5	5	5	5
Zeolite	4	3	2	1
Vit premix	1.5	1.5	1.5	1.5
Mineral premix	1.58	1.58	1.58	1.58
DL-Methionine	0.02	0.02	0.02	0.02
Coccidiostat	0.4	0.4	0.4	0.4
Herbal extract	0	1	2	3
Dried matter	88.88	89.45	90.16	90.84
Metabolized energy (Kcal g^−1^)	3.51	3.50	3.50	3.56
Protein (%)	44.82	44.90	44.93	45.01
Ether extract (%)	15.21	15.24	15.22	15.30
Crude fiber (%)	1.66	1.72	1.79	1.84
Total phosphorus (%)	1.30	1.30	1.30	1.30

**Table 2:** The growth performance of rainbow trout fed a diet supplemented with various concentrations of *Achillea wilhelmsii* (AW) for 8 weeks.

Treatments	Initial weight (g)	Final weight (g)	WG (g)	SGR	FCR
Control	11.6 ± 0.88	47.92 ± 0.74	36.30 ± 0.92	2.53 ± 0.12	1.16 ± 0.02
0.5% AW	11.4 ± 0.91	47.26 ± 2.20	35.84 ± 1.49	2.53 ± 0.08	1.18 ± 0.05
1% AW	11.3 ± 0.79	45.32 ± 3.00	33.94 ± 2.55	2.46 ± 0.10	1.25 ± 0.10
2% AW	11.9 ± 0.73	47.03 ± 4.76	35.10 ± 4.65	2.44 ± 0.19	1.22 ± 0.16

*Note*: Data are expressed as mean ± standard deviation. Significant differences between treatments are indicated by distinct superscript letters within the same row (*p*  < 0.05). The absence of superscripts denotes no significant differences between treatments.

Abbreviations: FCR, feed conversion ratio; SGR, specific growth rate; WG, weight gain.

**Table 3:** Hematological parameters of rainbow trout fed a diet enriched with various concentrations of *Achillea wilhelmsii* (AW) for 8 weeks.

Treatments	RBC (10^6^ mL^−1^)	WBC (10^3^ mL^−1^)	Htc (%)	Hb (gdL^−1^)	Lymphocytes (%)	Neutrophils (%)	Monocytes (%)	Eosinophils (%)
Control	1.71 ± 0.1^a^	13.80 ± 0.3^a^	38.34 ± 1.57^a^	6.55 ± 0.11^a^	80.60 ± 2.50^b^	17.60 ± 2.45^a^	1.6 ± 0.69	0.2 ± 0.42
0.5% AW	1.81 ± 0.03^b^	14.60 ± 0.8^ab^	39.96 ± 1.49^b^	7.16 ± 0.15^c^	79 ± 2.86^ab^	19 ± 3.01^ab^	1.4 ± 0.69	0.4 ± 0.51
1% AW	1.81 ± 0.09^b^	15.06 ± 1.38^b^	40.20 ± 1.99^b^	7.20 ± 0.24^c^	77 ± 1.94^a^	21.30 ± 2.21^b^	1.4 ± 0.84	0.4 ± 0.51
2% AW	1.80 ± 0.05^b^	15.38 ± 1.12^b^	40.99 ± 1.87^b^	6.82 ± 0.06^b^	79 ± 2.70^ab^	19 ± 2.53^ab^	1.6 ± 0.84	0.4 ± 0.51

*Note*: Data are presented as mean ± standard deviation. Significant differences between treatments are denoted by different superscript letters within the same row (*p*  < 0.05). The absence of superscripts indicates no significant differences between treatments.

Abbreviations: Hb, hemoglobin; Htc, hematocrit; RBC, red blood cell; WBC, white blood cell.

**Table 4:** Serum biochemical parameters of rainbow trout fed a diet enriched with various concentrations of *Achillea wilhelmsii* (AW) for 8 weeks.

Treatments	Glucose (mg dL^−1^)	Creatinine (mg dL^−1^)	AST (U dL^−1^)	ALT (U dL^−1^)	LDH (U dL^−1^)	Triglycerides (mg dL^−1^)	Cholesterol (mg dL^−1^)	TSP (g dL^−1^)
Control	50.49 ± 0.83	0.76 ± 0.1^b^	178 ± 7^ab^	0.75 ± 0.08^ab^	1648 ± 9^bc^	802 ± 14^b^	460 ± 8^c^	3.38 ± 0.53^a^
0.5% AW	49.80 ± 5.07	0.65 ± 0.14^b^	183 ± 39^b^	0.89 ± 0.32^b^	1703 ± 101^c^	909 ± 14^c^	357 ± 13^a^	3.47 ± 0.22^a^
1% AW	56.40 ± 16.73	0.51 ± 0.01^a^	156 ± 16^a^	0.59 ± 0.33^a^	1,391 ± 23^a^	650 ± 11^a^	361 ± 14^a^	3.64 ± 0.22^b^
2% AW	50.80 ± 7.14	0.72 ± 0.20^b^	196 ± 27^b^	0.94 ± 0.15^b^	1587 ± 89^b^	825 ± 50^b^	426 ± 10^b^	4.12 ± 0.17^c^

*Note*: The data are expressed as mean ± standard deviation. Significant differences between treatments are denoted by different superscript letters within the same row (*p*  < 0.05). If no superscripts are present, it indicates that there is no significant difference between treatments.

Abbreviations: ALP, alkaline phosphatase; AST, aspartate aminotransferase; LDH, lactate dehydrogenase; TSP, total serum protein.

**Table 5:** Serum immune parameters of rainbow trout fed a diet enriched with various concentrations of *Achillea wilhelmsii* (AW) for 8 weeks.

Treatments	IgM (g dL^−1^)	LYZ
Control	1.78 ± 0.05^ab^	1.88 ± 0.11^a^
0.5% AW	1.70 ± 0.09^a^	1.90 ± 10^a^
1% AW	1.81 ± 0.05^bc^	2.07 ± 0.17^b^
2% AW	1.89 ± 0.15^c^	2 ± 0.13^ab^

*Note:* Data are presented as mean ± standard deviation. Significant differences between treatments are shown by different superscript letters within the same row (*p*  < 0.05). The absence of superscripts indicates no significant difference between treatments.

Abbreviations: IgM, immunoglobulin M; LYZ, lysozyme.

**Table 6:** Serum bactericidal activity observed in fish fed varying levels of *Achillea wilhelmsii* (AW) for 8 weeks.

Bacterial pathogens	Treatments			
	Control	0.5% AW	1% AW	2% AW
*S. iniae*	4.95 ± 0.52	5.53 ± 0.68	5.51 ± 0.56	5.49 ± 0.63
*Y. ruckeri*	6.38 ± 0.32^ab^	6.21 ± 0.51^a^	6.69 ± 0.65^b^	7.25 ± 0.33^c^
*L. garviea*	7.96 ± 0.44	7.82 ± 1.02	8.09 ± 0.64	8.39 ± 0.29
*A. hydrophila*	5.77 ± 0.71^a^	6.09 ± 0.62^ab^	6.15 ± 0.56^ab^	6.48 ± 0.42^b^

*Note*: The data are presented as mean ± standard deviation. Significant differences between treatments are denoted by different superscript letters within the same row (*p*  < 0.05). The absence of superscripts indicates no significant difference between treatments.

## Data Availability

The data supporting the findings of this study are available from the corresponding author upon reasonable request.
